# Paradoxical effect of anti-inflammatory drugs on IL-6 mRNA expression in patients with PTSD during treatment

**DOI:** 10.1007/s00702-024-02770-6

**Published:** 2024-04-13

**Authors:** Cosima Rhein, Isabella Apelt, Franziska Werner, Eva Schäflein, Werner Adler, Martin Reichel, Caterina Schug, Eva Morawa, Yesim Erim

**Affiliations:** 1 Department of Psychosomatic Medicine and Psychotherapy, Friedrich-Alexander-Universität Erlangen-Nürnberg (FAU), Hartmannstr. 14, 91054 Erlangen, Germany; 2 Institute of Medical Informatics, Biometry and Epidemiology, Friedrich-Alexander-Universität Erlangen-Nürnberg (FAU), Erlangen, Germany; 3 Department of Psychiatry and Psychotherapy, Friedrich-Alexander-Universität Erlangen-Nürnberg (FAU), Erlangen, Germany; 4 Department of Nephrology and Medical Intensive Care, Charité-Universitätsmedizin Berlin, Berlin, Germany; 5 Department of Psychotherapy and Psychosomatic Medicine, Faculty of Medicine, Technische Universität Dresden (TUD), Dresden, Germany

**Keywords:** Posttraumatic stress disorder, Interleukin 6, Therapy outcome, Psychotherapy, Anti-inflammatory drugs

## Abstract

The pathophysiology of posttraumatic stress disorder (PTSD) is associated with the activation of the innate immune system, including cytokines like interleukin 6 (IL-6). However, the role of IL-6 in the etiology and treatment of PTSD still remains elusive. We conducted a prospective controlled trial to investigate the development of IL-6 during psychosomatic treatment in individuals with PTSD in comparison with individuals without PTSD. We assessed IL-6 mRNA expression before and after 2 months of psychosomatic treatment in individuals with and without PTSD. Severities of PTSD and depressive symptoms were assessed in parallel. Linear mixed regression was applied for statistical analysis, including the factors diagnosis PTSD and pre–post treatment after subgrouping for intake of anti-inflammatory drugs. The development of IL-6 mRNA expression during treatment was affected by the use of anti-inflammatory drugs. In the subgroup without intake of anti-inflammatory drugs, no significant statistical treatment effect in individuals with and without PTSD emerged. In the subgroup of individuals taking anti-inflammatory drugs, a significant interaction effect of the factors pre–post treatment and diagnosis PTSD was observed. Whereas IL-6 mRNA expression in individuals without PTSD decreased according to amelioration of symptoms, IL-6 mRNA expression in individuals with PTSD increased significantly during treatment, in opposite direction to symptom severity. Anti-inflammatory drugs might affect IL-6 mRNA expression in individuals with PTSD in a paradoxical way. This study offers a further piece of evidence that IL-6 could be involved in the pathophysiology of PTSD and PTSD-specific immunologic molecular mechanisms.

## Introduction

After potentially traumatic events, affected individuals can develop a posttraumatic stress disorder (PTSD). Core symptoms are avoidance behavior, hyperarousal, and intrusions/flashbacks. PTSD occurs with a lifetime prevalence of around 3.9% worldwide (Koenen et al. 2017). Suffering from PTSD increases the risk of cardiovascular and autoimmune diseases (Boscarino 2004; Kimerling 2004). Inflammation was suggested as a common pathway (Black 2006; Dennis et al. 2016). Specifically, low-grade subclinical inflammation could be a psychobiological mediator between the different symptoms (Speer et al. 2018).

Several studies have investigated immune activation in PTSD (Passos et al. 2015; Yang and Jiang 2020). Of highest interest are pro-inflammatory cytokines acting as immune modulating agents, which drive inflammatory states (Langgartner et al. 2018). Individuals with PTSD exhibit a general dysregulation of pathways regarding the innate immune system and inflammation (Hori et al. 2020). Meta-analyses indicate significantly higher levels of interferon gamma (IFN-γ), tumor necrosis factor-α (TNF-α), C-reactive protein (CRP), interleukin 1ß (IL-1ß) and interleukin 6 (IL-6) in individuals with PTSD compared with healthy controls (Passos et al. 2015; Yang and Jiang 2020). Studies showed that recovery from major depressive disorder (MDD) was paralleled by the reduction of initially increased cytokines (Dahl et al. 2016). Just few studies have been conducted to investigate the development of cytokines during PTSD treatment. However, these analyses could be helpful to see if treatment rebalances not only psychological symptoms, but also inflammatory states (Toft et al. 2018). Longitudinal studies focusing on pharmacological PTSD treatment have shown that paroxetine did not change IL-6 levels in cerebrospinal fluid in a mixed trauma population (Bonne et al. 2011). When citalopram or sertraline were used as treatment for PTSD, IL-1ß levels were reduced, and levels of its receptor, IL-2R, were increased during treatment (Tucker et al. 2004a). Few studies have investigated the development of cytokine levels during psychotherapeutic treatment. In one study, psychological distress after treatment decreased, indicated by the Global Severity Index (GSI), an overall scale taking into account various symptoms that are common to a heterogeneous psychiatric patient sample (Rytila-Manninen et al. 2016). Interestingly, cytokine levels increased, but only in individuals with PTSD (Toft et al. 2018). This effect was not detectable at the follow-up time point after 1 year, when cytokine levels did not differ between individuals with and without PTSD (Toft et al. 2021, 2022). Due to the important role of inflammation in PTSD, some studies included the use of anti-inflammatory drugs as covariates. There are different anti-inflammatory drugs aiming at counteracting inflammatory processes. Some of the most frequently used types of anti-inflammatory agents are non-steroidal anti-inflammatory drugs that inhibit prostaglandins, glucocorticoids that suppress cytokine-driven inflammation, and biologicals such as monoclonal antibodies (Dinarello 2010). One study showed that psychological distress decreased only in those inpatients that did not take anti-inflammatory drugs. Cytokine levels decreased in parallel and correlated with psychological distress after treatment. However, if patients used anti-inflammatory drugs, psychological distress was not reduced after treatment and cytokines did not decrease (Toft et al. 2020). The use of anti-inflammatory drugs was also associated with higher levels of cytokines (Toft et al. 2018).

In the present study, we focused on IL-6 levels, as IL-6 levels were reported to be significantly different between individuals with PTSD and healthy controls, and the highest number of well conducted studies exists for the cytokine IL-6 in PTSD (*n* = 15) (Passos et al. 2015). Moreover, we detected in an earlier study that IL-6 secretion after a stressful psychosocial intervention at the beginning of the therapy, namely the Trier Stress Test (TSST), predicted psychotherapeutic outcome in PTSD. High levels of IL-6 upon the TSST were associated with a negative therapy outcome (Rhein et al. 2021). Our results were consistent with Renner et al., who found that both pro- and anti-inflammatory IL-6 and IL-10 levels upon the TSST predicted therapy outcome of female individuals with PTSD. Whereas high IL-6 levels were predictive of more severe symptoms after therapy, high IL-10 levels were associated with a reduction of symptoms (Renner et al. 2022). Studies on the development process of IL-6 levels during psychosomatic treatment are scarce. Therefore, the aim of this explorative study was to investigate how IL-6 levels in individuals with and without PTSD develop during psychosomatic treatment, with the overall goal of identifying a biomarker for treatment monitoring. We hypothesized a decrease of IL-6 levels in the PTSD group upon treatment, in parallel with the amelioration of symptom severity.

## Materials and methods

### Study sample

We conducted an explorative study and asked all patients who were treated at the inpatient and daycare unit of the Department of Psychosomatic Medicine and Psychotherapy at the University Hospital Erlangen between September 2019 and March 2020 for participation. Inclusion criteria were age ≥ 18 years, sufficient German language proficiency, and regular admission for an inpatient or daycare unit treatment. Exclusion criteria were acute psychotic disorder, degenerative brain disorders like Alzheimer’s disease, and current substance dependency. In addition, we did not include patients with acute inflammation and infections such as SARS-CoV-2. This strategy resulted in the inclusion of 67 individuals (54 females, 13 males, average age 39.6 years ± 14.5, range from 21 to 67 years) (Table 1). The aim of the study was explained, and all included individuals gave their written consent to participate. During the first 2 days, a diagnostic interview was conducted by trained psychologists, and patients completed the psychometric assessments using an electronic system. Patients received psychosomatic treatment for 8 weeks including an adjustment of psychotropic drug administration if appropriate. Out of the patients, 12 received anti-inflammatory drugs (NSAIDs, corticosteroids, monoclonal antibodies, mesalazine) during the observation period (Table 1). At their last day at the hospital, patients again completed the psychometric assessment using the electronic system. Only those individuals who took part at the two time points were included in the analysis in order to provide longitudinal data. The study was approved by the Ethics Committee of the Friedrich-Alexander-University Erlangen-Nürnberg (FAU, ID 200_19 Bc) and conducted in concordance with the Declaration of Helsinki. Written informed consent was obtained from all participants.

**Table 1:** Clinical characteristics of included patients

Demography	
Number of patients	*N* = 67
Age (mean, SD)	39.6 (14.5)
Sex (% female)	80.6
Diagnosis PTSD/non-PTSD	32/35
Anti-inflammatory drugs	
NSAIDs	8
Corticosteroids	2
Monoclonal antibodies + corticosteroids	1
Monoclonal antibodies + mesalazine	1

*NSAIDs* nonsteroidal anti-inflammatory drugs

### Psychometric assessment

For obtaining a standardized psychiatric diagnosis at baseline, trained psychologists conducted the validated structured interview MINI-DIPS (*Diagnostisches Interview bei psychischen Störungen*, in German only) (Margraf et al. 2017). Symptoms of acute stress reaction or post-traumatic stress disorder were measured electronically by the German version of the PTSD Checklist for DSM-5 [PCL-5, applied without Life Events Checklist for DSM-5 (LEC-5); (Kruger-Gottschalk et al. 2017)] at baseline and after psychosomatic treatment. To assess the severity of depressive symptoms we conducted the German version of the PHQ-9, a subscale of the Patient Health Questionnaire [PHQ-D, *Gesundheitsfragebogen für Patienten*; (Löwe et al. 2002)] at baseline and after psychosomatic treatment using an electronic system.

### Psychosomatic treatment

During the multimodal group-based treatment for 8 weeks, techniques of both cognitive behavioral and psychodynamic psychotherapy were applied to all included patients. Weekly psychotherapeutic elements were psychotherapy in individual (75 min) and group (100–150 min) format, psychoeducation (0–50 min), skills training (50–125 min), mindfulness and relaxation methods (25–100 min), art therapy (0–120 min), concentrative movement therapy (0–120 min), and pharmacological therapy, if appropriate. The focus of treatment was set on stabilizing methods. Standardization and quality of treatment was controlled by weekly team meetings and internal and external supervision of the whole therapeutic team (Philipps et al. 2019).

### RNA, cDNA and quantitative PCR analysis

Blood samples for RNA extraction were drawn at baseline and after psychosomatic treatment in PAXgene TM Blood RNA tubes (Qiagen, Hilden, Germany) and stored at −80 °C. RNA was isolated using the PAXgene TM Blood RNA kit (Qiagen) according to the manufacturer’s instructions. The concentration of RNA was determined photometrically using a Nanodrop ND-1000 UV-Vis spectrophotometer (Thermo Scientific, Waltham, MA, USA). 200 ng of RNA were used in a 10-μL reverse transcription reaction using the qScript cDNA Synthesis Kit (QuantaBio, Beverly, MA, USA) to synthesize cDNA. Quantitative PCR was performed using a LightCycler 480 real-time PCR system (Roche, Mannheim, Germany) in SYBRgreen format. qPCR analyses were performed in a reaction volume of 10 µL containing 5 μL of FastStart Essential DNA Green Master Mix (Roche), primers at a final concentration of 10 µM each (Operon, Ebersberg, Germany), and 2.5 µL of diluted cDNA. The temperature profile used was 95 °C for 5 min followed by 40 cycles of amplification (95 °C for 10 s, 68 °C for 20 s, 72 °C for 30 s) with subsequent melting curve. The following primers were designed, tested and used: IL-6, forward 5′-cgagcccaccgggaacgaaa-3′, reverse 5′-tggaccgaaggcgcttgtgga-3′; HPRT, forward 5′-tccgcctcctcctctgctc-3′, reverse 5′-gaataaacaccctttccaaatcctca-3′. HPRT was earlier shown to serve as an appropriate reference gene in human blood samples (Rhein et al. 2012). Threshold cycles (Ct) were determined with the second-derivative maximum method, and relative mRNA expression levels were calculated with the 2^−ΔΔCt^ method (Schmittgen and Livak 2008) using LightCycler 480 software (release 1.5.0). mRNA expression levels were transformed to a logarithmic scale (log10).

### Statistical analysis

In three linear mixed regression models, we examined relationships between PTSD symptoms, depressive symptoms, and IL-6 mRNA expression, respectively, as dependent variables, and PTSD diagnosis and time (pre/post) as independent variables. Based on visual inspection of changes of the dependent variables between time points in the PTSD and non-PTSD groups, we decided to include an interaction term in the model for IL-6 but not for PTSD symptoms and depressive symptoms. When visualizing the additional influence of the use of anti-inflammatory drugs (yes/no) on IL-6 change, the interaction was present in the group where anti-inflammatory drugs were used, but not in the group, where they were not used. Therefore, we performed subgroup analysis (anti-inflammatory drug use yes/no) using mixed regression models where the association between IL-6 change and PTSD diagnosis and time was modeled with and without interaction between PTSD diagnosis and time, as suggested by graphical evidence. Regression coefficients and their 95% confidence intervals are provided, as well as partial Cohen's *f* squared as effect sizes. The significance level was set to 0.05. All statistical analyses were performed using R V 4.2.0 (R-Core-Team 2022) and IBM SPSS Statistics version 21. Mixed model regression was done in R using package lmerTest, Cohen's *f* squared was calculated using package effect size.

## Results

### Symptom severity decreases during treatment in individuals with and without PTSD

**Fig. 1:**
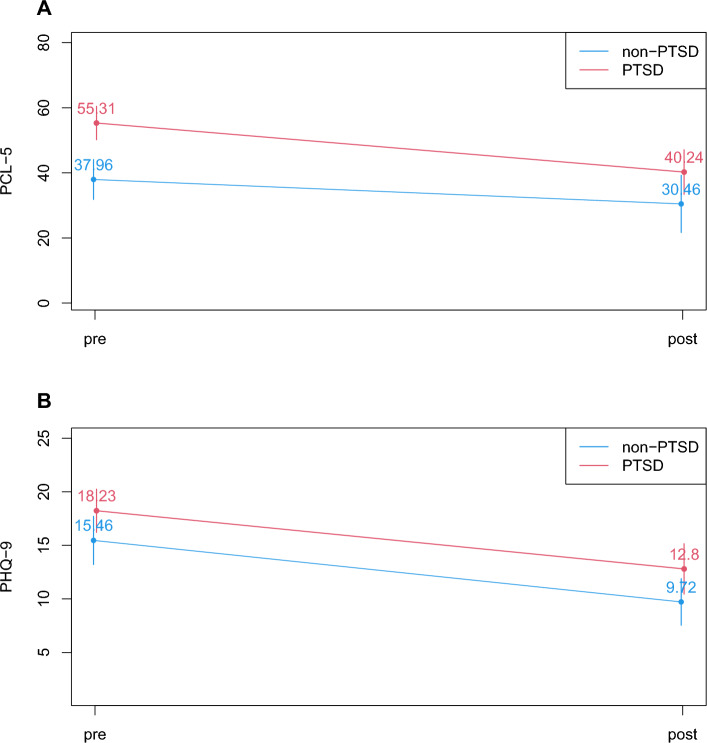
Symptom severity during treatment. **A** PTSD symptoms. PTSD symptoms were assessed using PCL-5 questionnaire before and after psychosomatic treatment. Both individuals with PTSD and individuals without PTSD showed significantly reduced symptom severity after treatment. Individuals with PTSD had significantly higher values in symptom severity compared with individuals without PTSD. **B** Depressive symptoms. Depressive symptoms were assessed using the PHQ-9 questionnaire before and after treatment. Both individuals with and individuals without PTSD showed significantly reduced depressive symptoms after treatment

After admission to the hospital, a standardized psychiatric diagnosis was obtained using the structured interview MINI-DIPS. Of the 67 patients included, *n* = 35 received the diagnosis PTSD, whereas patients with other diagnoses than PTSD were considered as the control group (*n* = 32). These diagnoses included depression (ICD-10: F31, F32, F33, F34), anxiety, phobia, compulsive disorder (ICD-10: F40, F41, F42), somatization (ICD-10: F45), and eating disorder (ICD-10: F50). All patients were diagnosed with more than one diagnosis. All patients received 8 weeks of multimodal group-based psychosomatic treatment with a focus on stabilizing methods. To evaluate the effect of therapeutic treatment on symptom severity, we compared the PCL-5 and PHQ-9 scores before and after treatment. A linear mixed regression analysis revealed that individuals with PTSD as well as individuals without PTSD showed significantly reduced PTSD symptom severity after treatment. Individuals with PTSD had significantly higher PTSD symptom severity before treatment compared with individuals without PTSD (Fig. 1A, Table 2). Both individuals with and without PTSD showed significantly reduced depressive symptoms after treatment (Fig. 1B, Table 2). Thus, psychosomatic treatment was successful and reduced symptom severity significantly independent of psychiatric diagnosis.

**Table 2:** Symptom severity during treatment

	Coefficient	95% CI		*p*	Cohen's *f*^2^
	PCL-5				
(Intercept)	39.868	33.373	46.363	**<0.001**	
Diagnosis PTSD (yes vs no)	13.697	4.972	22.423	**0.003**	0.190
Time (pre vs post treatment)	−11.749	−15.632	−7.866	**<0.001**	0.742
	PHQ-9				
(Intercept)	15.433	13.311	17.555	**<0.001**	
Diagnosis PTSD (yes vs no)	2.826	−0.020	5.672	0.052	0.080
Time (pre vs post treatment)	−5.694	−7.088	−4.300	**<0.001**	1.358

Significant results in bold, *p* < 0.05

PCL-5 assesses PTSD symptoms, PHQ-9 depressive symptoms. *CI* confidence interval

### Use of anti-inflammatory drugs and diagnosis affect development of IL-6 mRNA expression during treatment

**Fig. 2:**
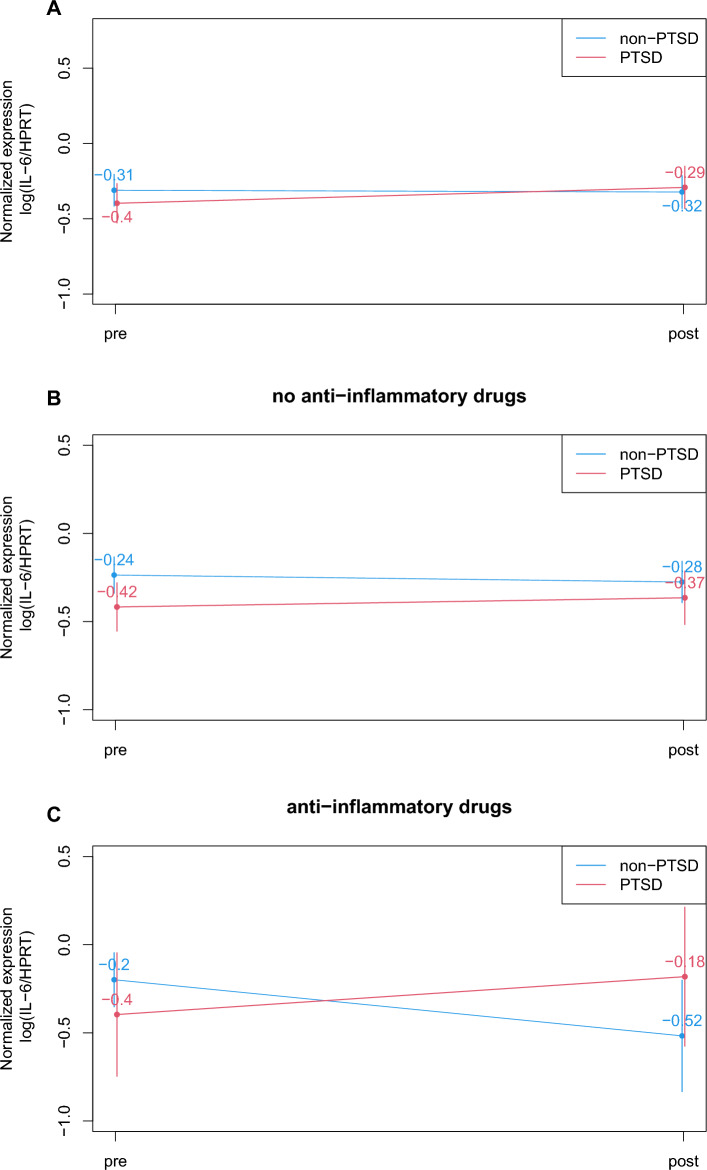
IL-6 mRNA expression during treatment. **A** Analysis without confounding factors. IL-6 mRNA expression levels were analyzed before and after treatment. IL-6 mRNA expression did not vary due to time (pre vs post treatment) or diagnosis (PTSD yes vs no). **B** Subgroup analysis: no use of anti-inflammatory drugs vs use of anti-inflammatory drugs. Both individuals with and individuals without PTSD not using anti-inflammatory drugs did not show changes in IL-6 mRNA expression levels during treatment. **C** For the group of patients using anti-inflammatory drugs during treatment, a significant interaction effect of time and diagnosis emerged. Individuals without PTSD showed a decrease, individuals with PTSD an increase in IL-6 mRNA expression levels during treatment

In parallel with psychometric assessment, IL-6 mRNA expression was analyzed in whole blood of patients before and after treatment. A linear mixed regression analysis showed no statistical effect of time or diagnosis on IL-6 mRNA expression in both individuals with and without PTSD (Fig. 2A, Table 3). However, taking into account the use of anti-inflammatory drugs revealed distinct effects. Individuals with and without PTSD not using anti-inflammatory drugs did not show changes in IL-6 mRNA expression levels during treatment, respectively (Fig. 2B, Table 3). In contrast, a significant interaction effect of time and diagnosis emerged for patients using anti-inflammatory drugs. Individuals without PTSD showed a decrease in IL-6 mRNA expression levels, whereas in individuals with PTSD, an increase in IL-6 mRNA expression was observed during treatment (Fig. 2C, Table 3). The use of anti-inflammatory drugs independent of diagnosis did not result in distinct effects on IL-6 mRNA expression or symptom severity reduction during treatment. Thus, the use of anti-inflammatory drugs in combination with psychiatric diagnosis seems to affect the development of IL-6 mRNA expression during treatment.

**Table 3:** Effects of PTSD diagnosis and anti-inflammatory drugs on IL-6 mRNA expression during treatment

	Coefficient	95% CI		*p*	Cohen's *f*^2^
	IL-6 expression of all included patients				
(Intercept)	−0.311	−0.429	−0.193	**<0.001**	
Diagnosis PTSD (yes vs no)	−0.086	−0.258	0.085	0.325	0.003
Time (pre vs post treatment)	−0.012	−0.16	0.137	0.878	0.013
Diagnosis × time	0.117	−0.098	0.332	0.292	0.021
	IL-6 expression of patients using anti-inflammatory drugs				
(Intercept)	−0.199	−0.626	0.229	0.382	
Diagnosis PTSD (yes vs no)	−0.197	−0.733	0.338	0.485	0.008
Time (pre vs post treatment)	−0.318	−0.588	−0.049	**0.046**	0.040
Diagnosis × time	0.533	0.195	0.871	**0.013**	1.062
	IL-6 expression of patients not using anti-inflammatory drugs				
(Intercept)	−0.257	−0.363	−0.151	**<0.001**	
Diagnosis PTSD (yes vs no)	−0.135	−0.263	−0.008	**0.044**	0.124
Time (pre vs post treatment)	0.003	−0.122	0.127	0.967	0

Significant results in bold, *p* < 0.05

*CI* confidence interval

## Discussion

The aim of this study was to investigate the development of IL-6 levels during psychosomatic treatment of individuals with PTSD and to identify modulating factors, with the overall goal of identifying a biomarker for treatment monitoring.

It turned out that it is difficult to determine IL-6 protein secretion at basal levels using ELISA (Rhein et al. 2021; Toft et al. 2022). Therefore, we decided to analyze IL-6 at the transcription state as mRNA expression to obtain more stable results. This procedure is supported by the review by Hori et al. (Hori et al. 2020), who investigated inflammatory markers in PTSD at the transcriptional level. Even though both protein secretion and mRNA expression measure different time points in IL-6 biology, both analyses add important data in the question of the role of IL-6 in PTSD. Compared with protein based studies (Toft et al. 2022), we did not have drop outs due to detection limits.

Severities of PTSD and depressive symptoms were found to decrease significantly during psychosomatic treatment independent of psychiatric diagnosis. This is in line with our earlier studies that showed that after psychosomatic treatment the greatest amelioration effects were detected regarding depressive symptoms (Kobel et al. 2020; Philipps et al. 2019). Other studies found PTSD symptoms to be decreased after psychosomatic treatment (Jergovic et al. 2015; Tucker et al. 2004b). Interestingly, these latter studies investigated the development of cytokines during treatment in parallel, and observed both increasing (Jergovic et al. 2015) and decreasing IL-1β levels (Tucker et al. 2004b). For TNF-α, increasing levels were reported in parallel with reduced symptom severity (Himmerich et al. 2016). Another study showed that whereas symptom severity decreased in individuals with PTSD, all investigated cytokines increased significantly after therapy (Toft et al. 2018). This was a highly unexpected result, and could be explained by the specific psychotherapeutic treatment that individuals with PTSD undergo. Re-experiencing traumatic situations during psychotherapy might result in stress reactions and subsequent immune activation (Toft et al. 2018). In addition, the well-described disruption of the hypothalamic-pituitary-adrenal (HPA) axis in PTSD could be responsible for dysregulated cytokine secretion (Raison and Miller 2003). Interestingly, our earlier study on group therapy in PTSD found an increase in somatic symptoms after therapy as measured by PHQ-15 (Philipps et al. 2019). Increased cytokine levels after treatment could be the corresponding molecules for this effect. However, in a further study by Toft et al., the group found a corresponding decline of psychological distress indicated by the Global Severity Index and cytokine levels only in those psychosomatic patients that did not take anti-inflammatory drugs (Toft et al. 2020). The overall levels of IL-1β, MCP-1, and TNF-α were higher in those individuals with PTSD who used anti-inflammatory drugs (Toft et al. 2018). This seems to be contradictory, as such drugs are used to decrease inflammatory processes. Specifically, IL-6 was shown to be inhibited by anti-inflammatory drugs (Kang et al. 2001; Tsuboi et al. 1995). Among patients not using anti-inflammatory drugs, individuals with PTSD showed significantly higher psychological distress compared with individuals without PTSD. This difference was not visible in those patients using anti-inflammatory drugs (Toft et al. 2020). Thus, anti-inflammatory drugs seem to influence the treatment outcome. Discussed as being important mediators in these studies (Toft et al. 2020), we included the use of anti-inflammatory drugs directly in our analyses. In line with studies by Toft et al., we found an increase in IL-6 levels in PTSD after treatment, despite a significant decrease in symptom severity. This effect occurred only in those individuals with PTSD taking anti-inflammatory drugs. In individuals without PTSD using anti-inflammatory drugs, a decrease in cytokine levels was observed, in parallel with decreased symptom severity. Thus, individuals with PTSD reacted differently from other psychosomatic patients who served as a control group in our study. In patients without anti-inflammatory drug usage, no effect on IL-6 during treatment was detected, but they also showed decreased symptom severity. Our results together with the work of Toft et al. point to a distinct immunologic response in individuals with PTSD upon therapy in contrast to other psychosomatic patients. Patients using anti-inflammatory drugs might show a reduced immunological response, but individuals with PTSD seem to react differently and might not benefit from these drugs (Toft et al. 2018). Other studies also suggest a distinct immunological response in PTSD (Gola et al. 2013). Anti-inflammatory drugs were suggested as being helpful in treating mood disorders, especially depressive disorders (Bai et al. 2020). However, our results do not point to the same effect for PTSD as seen for depressive disorders. Distinct molecular pathways of depressive symptoms could be responsible for different effects seen by the same treatment.

Limitations of this study are that we could include only few male patients, which excludes the analysis of sex effects. Due to the beginning of the COVID-19 pandemic in 2020, we had to stop recruiting patients and could not include a larger study population. The use of anti-inflammatory drugs was dependent on different indications (pain conditions vs autoimmune disease) and was assessed only once during the observation period. Undetected infections could have confounded results.

Altogether, there still remain obstacles to assessing the role of IL-6 in PTSD pathophysiology and etiology: the interaction of medication, the presence of comorbid psychiatric and somatic disorders, and possible research bias in the available studies (Nilsonne et al. 2016). More studies are needed to clarify the relationship and causal mechanisms between immune activation and PTSD before clinical trials can be started (Nilsonne et al. 2016). Future studies should investigate details of immunologic reaction including analysis of immune cell composition of the innate and adaptive immune system in individuals with PTSD during psychosomatic treatment.

## Data Availability

Data are available on request.
